# Oxygen delivery, oxygen consumption and decreased kidney function after cardiopulmonary bypass

**DOI:** 10.1371/journal.pone.0225541

**Published:** 2019-11-22

**Authors:** Rik H. J. Hendrix, Yuri M. Ganushchak, Patrick W. Weerwind

**Affiliations:** 1 Department of Extra-Corporeal Circulation, Maastricht University Medical Centre+, Maastricht, the Netherlands; 2 Cardiovascular Research Institute Maastricht (CARIM), Maastricht University, Maastricht, the Netherlands; University Medical Center Utrecht, NETHERLANDS

## Abstract

**Introduction:**

Low oxygen delivery during cardiopulmonary bypass is related to a range of adverse outcomes. Previous research specified certain critical oxygen delivery levels associated with acute kidney injury. However, a single universal critical oxygen delivery value is not sensible, as oxygen consumption has to be considered when determining critical delivery values. This study examined the associations between oxygen delivery and oxygen consumption and between oxygen delivery and kidney function in patients undergoing cardiopulmonary bypass.

**Methods:**

Oxygen delivery, oxygen consumption and kidney function decrease were retrospectively studied in 65 adult patients.

**Results:**

Mean oxygen consumption was 56 ± 8 ml/min/m^2^, mean oxygen delivery was 281 ± 39 ml/min/m^2^. Twenty-seven patients (42%) had an oxygen delivery lower than the previously mentioned critical value of 272 ml/min/m^2^. None of the patients developed acute kidney injury according to RIFLE criteria. However, in 10 patients (15%) a decrease in the estimated glomerular filtration rate of more than 10% was noted, which was not associated with oxygen delivery lower than 272 ml/min/m^2^. Eighteen patients had a strong correlation (r >0.500) between DO_2_ and VO_2_, but this was not related to low oxygen delivery. Central venous oxygen saturation (77 ± 3%), oxygen extraction ratio (21 ± 3%) and blood lactate levels at the end of surgery (1.2 ± 0.3 mmol/l) showed not to be indicative of insufficient oxygen delivery either.

**Conclusions:**

This study could not confirm an evident correlation between O_2_ delivery and O_2_ consumption or kidney function decrease, even at values below previously specified critical levels. The variability in O_2_ consumption however, is an indication that every patient has individual O_2_ needs, advocating for an individualized O_2_ delivery goal.

## Introduction

Oxygen delivery (DO_2_) during cardiopulmonary bypass (CPB) has received considerable attention in recent years, as it is one of the few modifiable factors related to acute kidney injury (AKI) and other morbidities. DO_2_ is dependent on hemoglobin concentration, oxygen saturation and blood flow. When either of these factors decreases the reduction is met by an increase in organ O_2_-extraction rate (OER) to meet metabolic needs, until the point of maximum O_2_-extraction. When DO_2_ falls beyond this point, referred to as the critical DO_2_ level (DO_2crit_), a pathologic supply dependency arises as organ oxygen consumption (VO_2_) decreases proportionally to decreasing DO_2_. At this point, cells enter the anaerobic metabolism phase and lactate levels rise [1–3].

Priming volume and crystalloid cardioplegic solution are the main contributors to a decreased hemoglobin concentration during CPB. The drop in oxygen carrying capacity due to hemodilution is often not considered when CPB flow rates are determined. The subsequent drop in DO_2_ does not have to be a problem however, as VO_2_ is also decreased by anaesthesia, possible hypothermia, and even by excluding and arresting the heart. This was rationalised by Ganushchak et al [4], who found no correlation between VO_2_ and DO_2_ during CPB. In contrast, other studies showed a correlation between perioperative DO_2_ and postoperative AKI, a severe complication with high morbidity and mortality, specifying DO_2crit_ levels in the range of 200–300 ml/min/m^2^ [5–7].

Since DO_2_ below a certain critical level can undoubtedly lead to a range of morbidities including AKI, goal directed perfusion strategies have aimed at keeping DO_2_ above critical levels. Even though this seems to decrease the rate of AKI [8, 9], performing CPB purely based on the goal of keeping DO_2_ above a universal value might not be the optimal perfusion strategy for every patient, as one may argue that using a single DO_2crit_ value for all patients is not sensible. DO_2crit_ cannot be considered without a patient’s VO_2_, which in turn depends on patient characteristics and is additionally influenced by factors like anaesthesia technique and temperature management, leading to different VO_2_ levels and subsequently different DO_2_ requirements for every patient. We therefore explored the relationship of DO_2_, VO_2_ and AKI in a group of patients undergoing CPB for cardiac surgery at our institution, and determined if one of the previously found DO_2crit_ levels can be applicable to all these patients as well.

## Methods

### Patients

A dataset of 67 adult patients who underwent elective CPB between May and November 2014 at the Maastricht University Medical Centre was retrospectively analysed in this study. Patients undergoing re-operations and procedures requiring deep hypothermic circulatory arrest were excluded from this study. This concerned 2 patients, resulting in data from 65 procedures. Data acquisition at the time and current data analyses were performed anonymously and included only routine measurements performed during CPB without the need for any intervention. In accordance with the Dutch law for approving medical research, Institutional Review Board approval was therefore waived.

### Anesthesia and perfusion techniques

General anaesthesia was induced using weight-related infusion of sufentanil (1.0 μg/kg), midazolam (0.1 mg/kg) and rocuronium bromide (1.0 mg/kg). Subsequently, maintenance doses of 5.0 mg/kg/h of propofol and 1.0 μg/kg/h of sufentanil were used. A Stöckert S5 heart-lung machine with a CP5 pump unit (LivaNova Deutschland GmbH, Munich, Germany) was used in all cases, and comprised a phosphorylcholine-coated adult oxygenator tubing pack (LivaNova, London, England) and a Revolution centrifugal pump (LivaNova). Before initiation of CPB the patient received a bolus (300 IU/kg body weight) of heparin (Leo Pharma B.V., Breda, Netherlands). CPB was started when activated clotting time (ACT, measured using HemoTec ACT II, Medtronic, Minneapolis, Minnesota, USA) was extended to at least 400 seconds. After central cannulation, pulsatile CPB (pulse frequency 70 bpm, pulse width 50%, base flow 30%) was started with a pump flow indexed at 2.4 l/min/m^2^. Arterial and venous blood gases were measured using the CDI500 blood parameter monitoring system (Terumo Corporation, Tokyo, Japan), and arterial pO_2_ was kept between 10–15 kPa as measured by CDI500. Mean arterial blood pressure was kept between 70 and 80 mmHg using phenylephrine titration when applicable. Cardiac arrest was induced using either cold crystalloid cardioplegia (St. Thomas II solution, Apotheek Haagse Ziekenhuizen, Den Haag, the Netherlands) or warm blood cardioplegia (pharmacy Catharina hospital, Eindhoven, the Netherlands). After cessation of CPB, protamine in a proportion of 1.0–1.2 mg/100 IU of the initial heparin dose was used as an antidote. If after this dose the ACT was still prolonged compared to baseline, the patient received an additional dose of protamine accordingly.

### Data collection and analysis

Continuous inline monitoring of arterial and venous blood gas parameters during cardiac arrest was done using the CDI500, which was calibrated according to the instructions for use. In addition, arterial and venous blood samples were sent to the laboratory when on full CPB support and before initiating weaning from CPB. Laboratory results were used to recalculate the CDI500 data for hemoglobin towards more accurate results by means of regression. These, and all other CPB data were digitally gathered in a patient data management system (PDMS, Chipsoft B.V., Amsterdam, the Netherlands). All data were automatically synchronized by the PDMS and combined into one output file at a rate of 6 data points per minute.

DO_2_ and VO_2_ were calculated using recalculated CDI data as follows:

$${\mathrm{DO}}_{2}\;({\mathrm{ml}/\min/m}^{2})={\mathrm{CaO}}_{2}\times Q_{b}$$

$${\mathrm{VO}}_{2}({\mathrm{ml}/\min/m}^{2})=({\mathrm{CaO}}_{2}‐{\mathrm{CvO}}_{2})\times Q_{b}$$

Where Q_b_ is blood flow in dl/min/m^2^ body surface area (BSA) and CaO_2_ and CvO_2_ are arterial and venous oxygen content respectively (in ml/dl). The latter were calculated using the following equations:

$${\mathrm{CaO}}_{2}\;(\mathrm{ml}/\mathrm{dl})=(\left(\mathrm{hb}\times 1.36\times {\mathrm{SaO}}_{2})+({\mathrm{PaO}}_{2}\times 0.003\right))$$

$${\mathrm{CvO}}_{2}\;(\mathrm{ml}/\mathrm{dl})=(\left(\mathrm{hb}\times 1.36\times {\mathrm{SvO}}_{2})+({\mathrm{PvO}}_{2}\times 0.003\right))$$

Where hb is hemoglobin concentration in g/dl; SaO_2_ and SvO_2_ are arterial and venous O_2_ saturations (decimal) and PaO_2_ and PvO_2_ are the partial pressures of O_2_ in arterial and venous blood respectively (in mmHg).

The oxygen extraction ratio (OER) was calculated by dividing VO_2_ and DO_2_:

$$\mathrm{OER}\;(\%)=\left(\frac{{\mathrm{VO}}_{2}}{{\mathrm{DO}}_{2}}\right)\times 100$$

Where both VO_2_ and DO_2_ are in ml/dl/m^2^.

As most perfusion goals are aimed at keeping DO_2_ above 270–300 ml/min/m^2^ [8, 9] we chose to test if the DO_2crit_ level found by Ranucci et al [6] (272 ml/min/m^2^) applies to our patient population. Therefore, the population was split in a group with a mean DO_2_ higher than 272 ml/min/m^2^ during the aortic cross clamp time (group DO_2_ >272) and a group with a mean DO_2_ lower than 272 ml/min/m^2^ (group DO_2_ <272). The two groups were then compared on the prevalence of postoperative kidney function decrease, which was assessed by calculating the estimated glomerular filtration rate (eGFR) using the simplified 'modification of diet in renal disease' formula [10],

$$\mathrm{eGFR}\;(\mathrm{mL}/\min/1.73\;m^{2})=186\times {\left(S_{\mathrm{cr}}\right)}^{‐1.154}\times {\left(A\right)}^{‐0.203}\times (0.742\;\mathrm{if}\;\mathrm{female})\times (1.210\;\mathrm{if}\;\mathrm{black})$$

Where S_cr_ is the serum creatinine level in mg/dl and A is the age in years. According to RIFLE criteria, patients in the lowest AKI class have a more than 25% decrease in eGFR persisting >24h [11, 12]. However, our study population contained no patients meeting these criteria. To still be able to evaluate the relationship between DO_2_ and kidney function decrease in this population, we defined a 10% decrease in eGFR (ΔeGFR) compared to the preoperative value as declined postoperative kidney function.

Patients were divided into a group with >10% kidney function decline and a group with less decline or no decline at all. Differences between these groups were then analysed.

Statistical analysis was performed using IBM SPSS, version 23. Data are presented as mean ± standard deviation (sd) where applicable. To compare the DO_2_ and ΔeGFR groups on pre-, intra- and postoperative characteristics, Mann-Whitney U tests and Chi-squared tests were performed. Analysis of the relation between DO_2_ and ΔeGFR was done by crosstab analysis with the exact test module [13, 14] and a receiver operating characteristic curve. Oldham correlation analysis was performed to look for relations between DO_2_ and VO_2_ per patient, and crosstab analysis with an exact test was used to analyse the effect of low DO_2_ on these correlations. Blood lactate levels at the start of CPB and at the end of CPB were compared with a paired sample t-test.

## Results

The majority of the patients (n = 48) included in this study underwent isolated coronary artery bypass grafting (CABG) or isolated aortic valve replacement (AVR, n = 13). One case was a combined intervention (CABG + AVR) and one case was a mitral valve plasty (MVP). All these procedures were performed under normothermic CPB. Additionally, two procedures were performed using mild hypothermia (one Bentall procedure at 32 degrees Celsius, and one combined MVP + CABG at 34 degrees Celsius).

Mean VO_2_ was 56 ± 8 ml/min/m^2^, varying between 38 and 76 ml/min/m^2^, whereas mean OER was 20 ± 3%. Mean oxygen delivery was 281 ± 39 ml/min/m^2^, ranging from 181 ml/min/m^2^ to 415 ml/min/m^2^. Twenty-seven patients (42%) had an average DO_2_ <272 ml/min/m^2^, the other 38 patients (58%) had an average DO_2_ >272 ml/min/m^2^. The preoperative EuroSCORE II and the percentage of patients with diabetes were significantly higher in the DO_2_ <272 group (Table 1), whereas both preoperative and intraoperative hemoglobin levels were significantly higher in the DO_2_ >272 group. Even though values were still well within the normal physiologic ranges, patients in the DO_2_ <272 group had a significantly lower PvO_2_ and SvO_2_. Intraoperative VO_2_ and OER did not differ between the low and high DO_2_ groups, nor were there any significant differences in postoperative eGFR, the percentage of patients with a ΔeGFR >10% or postoperative lactate levels.

**Table 1:** Pre-, intra- and postoperative characteristics split on average DO_2_.

	DO_2_ <272 n = 27	DO_2_ >272 n = 38	p-value
**Preoperative**			
Age (years)	68 ± 11	67 ± 9	0.399
Gender male (% (n))	70 (19)	92 (35)	0.041
Height (cm)	171 ± 9	173 ± 7	0.395
Weight (kg)	86 ± 15	83 ± 14	0.354
BSA (m^2^)	2.0 ± 0.2	2.0 ± 0.2	0.659
Diabetes mellitus II (% (n))	48% (10)	23% (10)	0.042
Hypertension (% (n))	100% (27)	100% (38)	-
Mean Euroscore II ± SD	3.1 ± 2.6	1.7 ± 1.5	0.035
LVEF (%, median (IQR))	55 (45–58)	55 (51–60)	0.374
eGFR (ml/min/1.73m^2^)	76 ± 23	75 ± 17	0.674
Hemoglobin (mmol/l)	8.2 ± 0.9	8.7 ± 0.8	0.012
Lactate (mmol/l)	0.9 ± 0.3	1.0 ± 0.3	0.070
**Intraoperative**			
Hemoglobin (mmol/l)	4.5 ± 0.4	5.4 ± 0.5	<0.001
Blood flow (l/min)	5.1 ± 0.6	5.4 ± 0.3	0.197
Sweep gas flow (l/min)	2.4 ± 0.5	2.6 ± 0.5	0.176
FiO_2_ (%)	0.47 ± 0.05	0.47 ± 0.04	0.424
PaO_2_ (kPa)	13.1 ± 2.0	12.7 ± 1.8	0.161
PvO_2_ (kPa)	4.9 ± 0.4	5.2 ± 0.4	0.032
SaO_2_ (%)	97 ± 1	97 ± 1	0.614
SvO_2_ (%)	75 ± 4	78 ± 3	0.005
DO_2_ (ml/min/m^2^)	247 ± 22	308 ± 28	<0.001
VO_2_ (ml/min/m^2^)	58 ± 10	62 ± 7	0.082
OER (%)	22 ± 4	21 ± 3	0.217
MAP (mmHg)	70 ± 7	71 ± 5	0.546
ECC time (min)	98 ± 41	99 ± 34	0.623
Aortic occlusion time (min)	63 ± 27	67 ± 21	0.380
**Postoperative**			
Lactate (mmol/l)	1.2 ± 0.4	1.2 ± 0.3	0.880
eGFR (ml/min/1.73m^2^)	81 ± 26	80 ± 23	0.584
ΔeGFR >10% (% (n))	14 (3)	16 (7)	0.865

BSA—body surface area; LVEF—left ventricular ejection fraction; IQR—interquartile range; FiO_2_—fraction of O_2_ in sweep gas; PaO_2_ and PvO_2_—arterial and venous partial O_2_ pressures; SaO_2_ and SvO_2_—arterial and venous O_2_ saturations; DO_2_—O_2_ delivery; OER—O_2_ extraction ratio; ABP—arterial blood pressure; ECC—extracorporeal circulation; eGFR—estimated glomerular filtration rate.

The correlation between DO_2_ and VO_2_ was strong (r >0.500) in 18 patients, of whom 5 patients were in the DO_2_ <272 group. Crosstab analysis showed that DO_2_ <272 was not related to having a correlation between DO_2_ and VO_2_ (p = 0.260).

**Fig 1:**
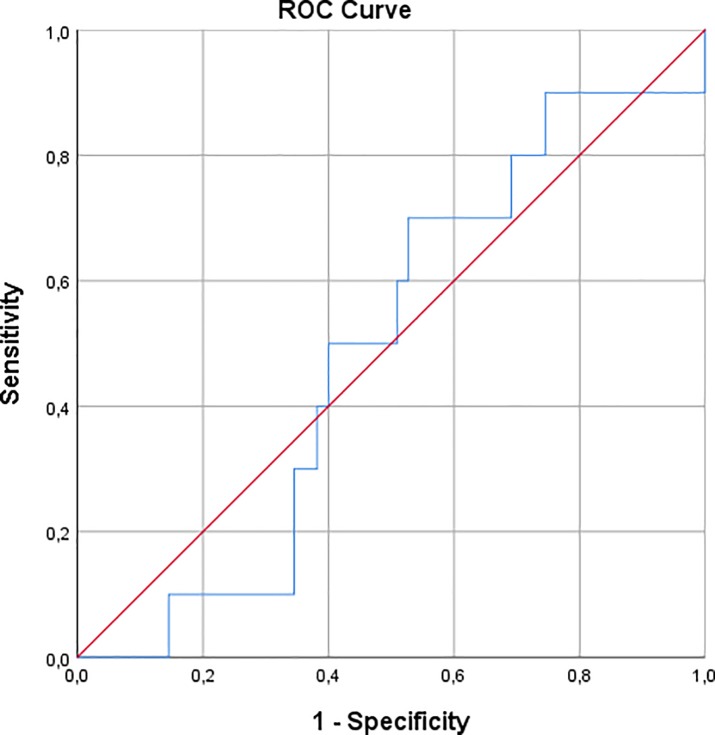
Receiver operating characteristic curve of DO_2_ and ΔeGFR >10%. DO_2_ –oxygen delivery; ΔeGFR >10%—more than 10% decrease in estimated glomerular filtration rate.

When split on a ΔeGFR of 10%, fifty-five patients (85%) had a ΔeGFR <10%, whereas 10 patients (15%) had a ΔeGFR >10%. Crosstab analysis showed no significant differences between the two DO_2_ groups concerning the percentage of patients with a ΔeGFR >10% (p = 0.865, Table 2). Moreover, a receiver operating characteristic analysis showed an area under the curve of 0.491 (Fig 1), indicating that DO_2_ did not predict ΔeGFR >10%.

Comparing the two ΔeGFR groups in terms of other patient and CPB characteristics (Table 3) revealed that patients in the ΔeGFR >10% group had a significantly lower pre-operative hemoglobin level. Intraoperatively however, there was no significant difference in the hemoglobin level. DO_2_, VO_2_ and OER did not significantly differ between the ΔeGFR groups.

**Table 2:** Crosstab analysis of the relation between both DO_2_ groups and ΔeGFR groups.

	ΔeGFR <10%	ΔeGFR >10%	p-value
DO_2_ <272 (n)	24 (89%)	3 (11%)	0.503
DO_2_ >272 (n)	31 (79%)	7 (21%)	

DO_2_—oxygen delivery; ΔeGFR–decrease in estimated glomerular filtration rate.

**Table 3:** Preoperative and intraoperative variables split based on a ΔeGFR of 10%.

	ΔGFR <10% (n = 55)	ΔGFR >10% (n = 10)	p-value
Preoperative			
Age (years)	68 ± 9	64 ± 10	0.193
Height (cm)	172 ± 8	172 ± 6	0.778
Weight (kg)	84 ± 15	87 ± 14	0.611
BSA (m^2^)	1.97 ± 0.19	2.00 ± 0.15	0.669
Diabetes mellitus II (% (n))	31 (17)	30 (3)	0.954
Hypertension (% (n))	100 (55)	100 (10)	-
Mean euroscore II ± SD	2.1 ± 2.0	2.8 ± 2.3	0.271
LVEF (median (IQR))	55 (50–60)	55 (43–55)	0.144
Hemoglobin (mmol/l)	8.6 ± 0.8	8.1 ± 0.7	0.032
Urea (mmol/L)	5.7 ± 2.1	7.1 ± 2.7	0.073
Creatinine (mmol/L)	92 ± 21	101 ± 22	0.244
eGFR (mL/min/1.73 m^2^)	76 ± 19	69 ± 20	0.178
Intraoperative			
Hemoglobin (mmol/l)	5.1 ± 0.6	4.9 ± 0.7	0.525
Blood flow (l/min)	5.2 ± 0.5	5.4 ± 0.5	0.393
Sweep gas flow (l/min)	2.5 ± 0.5	2.6 ± 0.6	0.716
FiO_2_ (%)	0.47 ± 0.04	0.49 ± 0.05	0.078
DO_2_ (ml/min/m^2^)	289 ± 39	283 ± 40	0.913
VO_2_ (ml/min/m^2^)	56 ± 9	56 ± 8	0.824
OER (%)	20 ± 3	21 ± 3	0.818
MAP (mmHg)	71 ± 6	71 ± 6	0.585
CPB time (min)	94 ± 27	127 ± 62	0.110
Aortic occlusion time (min)	64 ± 20	79 ± 34	0.252
Postoperative			
Urea (mmol/L)	5.0 ± 1.6	7.6 ± 2.9	0.004
Creatinine (mmol/L)	85 ± 23	119 ± 27	<0.001
eGFR (mL/min/1.73 m^2^)	85 ± 23	57 ± 19	0.001

BSA—body surface area; LVEF—left ventricular ejection fraction; IQR—interquartile range; eGFR—estimated glomerular filtration rate; FiO_2_—fraction of O_2_ in sweep gas; DO_2_—O_2_ delivery; VO_2_—O_2_ consumption; OER—O_2_ extraction ratio; MAP—mean arterial pressure; CPB—cardiopulmonary bypass.

## Discussion

This study was intended to explore the relationship between DO_2_ and VO_2_ and AKI in patients undergoing CPB for cardiac surgery at our institution and to verify if the previously found DO_2crit_ of 272 ml/min/m^2^ was applicable. Twenty-seven patients (42%) had an average DO_2_ lower than the DO_2crit_ of 272 ml/min/m^2^, caused by a significantly lower perioperative hemoglobin level. Even though DO_2_ in this group was lower than the proposed critical DO_2_ value of 272 ml/min/m^2^, there were no patients with AKI according to the RIFLE criteria (>25% decrease in eGFR). To still be able to analyse if the low DO_2_ led to decreased kidney function, we chose to define decreased kidney function as a more than 10% decrease in eGFR compared to the preoperative value. Whether this decrease is clinically relevant, needs to be further determined. Previous research, however, has shown that even small decreases in eGFR and/or small increases in serum creatinine, and even detection of renal injury markers without actual renal function decrease are related to increased morbidity and mortality [15]. Other research, however, has indicated that serum markers might not be reliable during hemodilution [16]. There were 10 patients (17%) with a ΔeGFR >10%, but there was no association between this decreased kidney function and low DO_2_.

When analysing the VO_2_/DO_2_ relationship per individual patient, 18 patients had a positive correlation between DO_2_ and VO_2_. This however, showed not to be related to DO_2_ <272 ml/min/m^2^. Moreover, other parameters supported that the positive correlation between DO_2_ and VO_2_ in these patients did not arise because of pathologic supply dependency and that DO_2_ was adequate for aerobic metabolism. Central venous oxygen saturation (SvO_2_) is a routinely used marker of systemic tissue perfusion, reflecting the balance between DO_2_ and VO_2_. In our study population SvO_2_ was 77 ± 3%, which is higher than associated with tissue hypoxia and increased postoperative morbidity and mortality [17]. The OER of 21 ± 3% was in the normal range, indicating sufficient DO_2_ as well. Furthermore, the lactate level before weaning (1.2 ± 0.3 mmol/l) was in the physiologic range, after a clinically not relevant increase from 1.0 ± 0.3 mmol/l at the start of CPB. A possible explanation for this trivial increase in lactate levels prior to CPB cessation might be washout of lactate accumulated in the heart during the aortic occlusion time. All this indicates that, despite mostly normothermic CPB and “low” DO_2_ in 42% of the patient population, DO_2_ seems to have been sufficient to prevent kidney function decrease or the commence of anaerobic metabolism.

Analysing the differences between the two ΔeGFR groups, we found that all the tested parameters were similar, except for the preoperative hemoglobin level. This value however (8.6 ± 0.8 mmol/l in the ΔGFR <10% group and 8.1 ± 0.7 mmol/l in the ΔGFR >10% group), was still well above anaemia levels associated with increased risk of AKI [18]. Moreover, the perioperative hemoglobin levels were similar between groups. The lack of differences between the two groups could be a result of the defined ΔeGFR of 10% which was necessary as the study population contained no patients with AKI according to RIFLE criteria. Some factors that may have contributed to the absence of AKI are the relatively high perioperative mean arterial blood pressure (70 ± 7 mmHg and 71 ± 5 mmHg in the low and high DO_2_ groups, respectively), which was similar to the optimal blood pressure during CPB found by Hori et al. [19] and the routine use of pulsatile blood flow that might be protective of renal function [20]. In addition, the patients scored relatively low at some AKI risk factors e.g.: no emergent surgery or patients with cardiogenic shock, 17% female gender; 8% COPD; 5% LVEF <35%; 17% peripheral vascular disease; 18% preoperative renal insufficiency (GFR <60 ml/min/m^2^) [21]. Whether the average CPB and aortic occlusion time of 99 ± 36 minutes and 66 ± 23 minutes respectively increase or decrease the risk of AKI is not clear, as some studies found that a CPB duration longer than 60 or 90 minutes increases the risk of AKI, whereas others compared AKI patients to non-AKI patients and found that the 100 minute CPB duration of the non-AKI group was lower than the CPB duration in the AKI group [22–25]. An explanation for the higher kidney function decrease in the ΔeGFR >10% group might be the higher incidence of diabetes, a known risk factor for AKI [21, 26].

We were not able to calculate DO_2crit_ levels for patients in this study, as we did not find an association between DO_2_ and kidney function decrease, nor did we find an indication of the onset of the pathologic supply dependency between DO_2_ and VO_2_. The large range of VO_2_ values (ranging from 38 to 76 ml/min/m^2^) makes clear though, that it is a highly variable factor depending on many elements and that therefore, a perfusion goal targeting a generic pre-established value for DO_2_ just above a general DO_2crit_ level cannot guarantee appropriate DO_2_ for all patients at all times. Indexing DO_2_ to patient body surface area (as is done for DO_2crit_ levels) allows for differences in physique, but does not take into account e.g. differences in gender and age. Furthermore, the oxygen necessity during CPB depends not only on patient characteristics, but also on factors like anaesthesia technique and temperature management. Thus, when targeting or studying DO_2_, VO_2_ should always be taken into account.

This study was solely designed to look for the relationship between DO_2_, VO_2_ and kidney function in patients undergoing CPB at our institution, as we questioned the usefulness of universal DO_2crit_ levels from previous research. The limited database compared to the large study populations in previous studies [5–7] resulted in a small sample size. Nevertheless, with research suggesting an incidence of AKI up to 40% after cardiac surgery (depending on the type of surgical procedure and the used definition of AKI) [27–29], we expected to have some patients in our population developing AKI, but the absence thereof resulted in the inability to determine patient specific DO_2crit_ values.

In conclusion, this study could not confirm an evident correlation between low O_2_ delivery and kidney function decrease in patients undergoing CPB for cardiac surgery. In addition, there was no indication for the presence of a pathologic supply dependency between DO_2_ and VO_2_. The variability in O_2_ consumption, however, is an indication that every patient has individual O_2_ needs, advocating for an individualized O_2_ delivery goal instead of a generic DO_2crit_ level.
